# Editorial: Molecular and cellular mechanisms in preimplantation IVF-embryo development

**DOI:** 10.3389/fcell.2023.1279129

**Published:** 2023-08-31

**Authors:** Renee J. Chosed, Shahryar K. Kavoussi, Dara S. Berger, Kaylee Massman, Maria Guerra-Velasquez

**Affiliations:** ^1^ Department of Biomedical Sciences, University of South Carolina School of Medicine Greenville, Greenville, SC, United States; ^2^ Austin Fertility and Reproductive Medicine/Westlake IVF, Austin, TX, United States; ^3^ Department of Obstetrics and Gynecology, University of Pennsylvania, Philadelphia, PA, United States

**Keywords:** blastocyst, preimplantation embryo, IVF, embryo development, PGT-A

The aims of this Frontiers Research Topic were to further our understanding of how the preimplantation embryo develops, and to consider current techniques used to evaluate embryo viability. If we can deepen our understanding of the molecular and cellular events taking place during preimplantation embryo development, we will be closer to identifying new potential molecular markers of embryo viability, which may one day be used to increase the success of *in vitro* fertilization (IVF).

Much of what is known about human embryo development at the cell and molecular level was discovered in mouse embryos or other animal models. Therefore, understanding the similarities and differences between human embryo development and other animal models is essential in clarifying the molecular steps taking place during human embryo development (reviewed by Bissiere et al., 2023). Researchers often rely on cultured cell lines and stem cells to understand these molecular and cellular events (White and Plachta, 2020). For example, Oh et al. report deriving lineage specific embryonic cell lines from porcine blastocysts to assess gene expression.

For patients undergoing IVF, the embryo selection process for transfer has traditionally relied on morphology metrics, and more recently, genetic analysis of cells, such as preimplantation genetic testing for aneuploidy (PGT-A) from the trophectoderm (reviewed by Harris et al., 2021). Interestingly, advancements in imaging and specifically time-lapse imaging throughout the culture period of preimplantation embryos, have provided new avenues for morphology studies. For example, several recent reports on the presence of cytoplasmic strings (via time-lapse imaging) in preimplantation embryos conceived via IVF have been published that try to link this morphological structure with blastocyst quality and pregnancy outcomes (Ma et al., 2022; Joo et al., 2023). Arguably the most cutting-edge research associated with embryo time-lapse imaging is the application of artificial intelligence (AI) for embryo screening. Using AI as a means of non-invasive embryo screening is a promising area of research yet there is still more to learn before we will see AI routinely used in the clinic (reviewed by Jiang and Bormann, 2023).

Some efforts have focused on using molecular markers from spent media and blastocoel fluid to assess the viability of the preimplantation embryo. Cell-free DNA and the contents of exosomes found in spent embryo culture media have all been studied to try and understand their specific origin from within the preimplantation embryo, and then attempting to clarify what these molecules tell us about the developing embryo (reviewed by Handayani et al., 2023; Saadeldin et al., 2023). The origin of cell-free DNA in the spent media and blastocoel fluid is thought to be from aneuploid cells that underwent apoptosis, yet there has been extensive research to use this cell-free DNA (non-invasive PGT-A) to assess embryo ploidy status that has yielded mixed results (reviewed by García-Pascual et al., 2023; Cinnioglu et al., 2023). Other molecules in the spent embryo culture media can also be detected and potentially serve as markers for embryo viability. For example, Meng et al. report the use of a Raman spectroscopy method to assess the metabolome present in spent embryonic culture media. However, there is still much that is unknown regarding preimplantation embryonic development and measurable indicators of successful embryo transfer.

Xu et al. reported the identification of two genetic variants of the *PADI6* gene in two patients who experienced infertility. The PADI6 protein is part of a multi-protein complex that is required for embryo development, specifically zygote gene activation. While this study used a very small sample size, it still highlights the need for further identification of genetic mutations that are associated with genes regulating embryo development. Therefore, identified mutations in a patients may allow for different counseling for women seeking Assisted Reproductive Technologies (ART), as these mutations may predict poor IVF outcomes. If specific poor-IVF-outcome-gene-mutations can be identified, then a future step could be finding ways to alter/fix these mutations in the preimplantation embryo. Yaghoobi et al. propose a strategy to take the molecular knowledge we have regarding embryo implantation to alter gene expression in the trophoblast layer of the developing blastocyst via CRISPR/dCas9 exosome system.

Having a more thorough understanding of the changes taking place in this stage of development may allow for better use and interpretation of biomarkers of embryo viability, therefore increasing IVF success rates.
